# A scoping review of social determinants of health contributing to disparities in dementia‐associated variables in Arabic populations

**DOI:** 10.1002/alz70858_102007

**Published:** 2025-12-25

**Authors:** Mohamed Taiebine, Saadia Aidi

**Affiliations:** ^1^ Euro‐Mediterranean University of Fez (UEMF), Fez, Fez, Morocco; ^2^ Department of Neurology and Neuropsychology, Specialty Hospital, Rabat, Morocco; ^3^ Faculty of Medicine and Pharmacy, University Mohammed V, Rabat, Morocco

## Abstract

**Background:**

Dementia, an umbrella syndrome encompassing various neurodegenerative diseases, disproportionately affects certain populations. Social determinants of health (SDOH) are defined as non‐medical factors that significantly influence an individual's health status and outcome. It's a result of a complex interplay of micro‐, meso‐ and macro‐level factors. This study investigates how SDOH contribute to disparities in dementia‐associated variables among Arabic populations in North‐African and Middle East (MENA) region.

**Method:**

A systematic scoping review was conducted via PubMed, Scopus and Web of Science databases following frameworks described by Arksey and O’Malley (2007) for articles published in English that addressed SDOH contributing to disparities in dementia‐associated variables. A summary of demographic and existing SDOH factors is included to assess each factor related to dementia in MENA region.

**Result:**

A total of 63 articles were identified through search engines and 20 duplicates were excluded. After title and abstract screening, another 36 studies were removed. Finally, a total of 7 full‐text studies were selected. This review reveals association between specific SDOH factors and variations in dementia risk, age of onset, and disease progression within Arabic populations. Furthermore, family cohesion, socio‐economic, literacy and access to health can influence dementia risk factors which are unique to Arabic context. They might interact with meso and macro‐structural factors which could influence caregiving dynamics and access to support.

**Conclusion:**

The paucity of studies reflects a need for future research of social SDOH related to dementia in Arabic populations. Understanding how SDOH contribute to dementia disparities is crucial for informing culturally appropriate interventions and effective policymaking strategies.

